# Sample Preservation, DNA or RNA Extraction and Data Analysis for High-Throughput Phytoplankton Community Sequencing

**DOI:** 10.3389/fmicb.2017.01848

**Published:** 2017-09-26

**Authors:** Anita Mäki, Pauliina Salmi, Anu Mikkonen, Anke Kremp, Marja Tiirola

**Affiliations:** ^1^Department of Biological and Environmental Science, University of Jyväskylä, Jyväskylä, Finland; ^2^Marine Research Centre, Finnish Environment Institute, Helsinki, Finland

**Keywords:** next generation sequencing, phytoplankton, cell lysis, operational taxonomic units, Lugol

## Abstract

Phytoplankton is the basis for aquatic food webs and mirrors the water quality. Conventionally, phytoplankton analysis has been done using time consuming and partly subjective microscopic observations, but next generation sequencing (NGS) technologies provide promising potential for rapid automated examination of environmental samples. Because many phytoplankton species have tough cell walls, methods for cell lysis and DNA or RNA isolation need to be efficient to allow unbiased nucleic acid retrieval. Here, we analyzed how two phytoplankton preservation methods, three commercial DNA extraction kits and their improvements, three RNA extraction methods, and two data analysis procedures affected the results of the NGS analysis. A mock community was pooled from phytoplankton species with variation in nucleus size and cell wall hardness. Although the study showed potential for studying Lugol-preserved sample collections, it demonstrated critical challenges in the DNA-based phytoplankton analysis in overall. The 18S rRNA gene sequencing output was highly affected by the variation in the rRNA gene copy numbers per cell, while sample preservation and nucleic acid extraction methods formed another source of variation. At the top, sequence-specific variation in the data quality introduced unexpected bioinformatics bias when the sliding-window method was used for the quality trimming of the Ion Torrent data. While DNA-based analyses did not correlate with biomasses or cell numbers of the mock community, rRNA-based analyses were less affected by different RNA extraction procedures and had better match with the biomasses, dry weight and carbon contents, and are therefore recommended for quantitative phytoplankton analyses.

## Introduction

Phytoplankton is often used to monitor the status of aquatic ecosystems, and effective methods for the characterization of phytoplankton samples are needed. Traditionally, phytoplankton community compositions have been studied using microscopic techniques and observing morphological characteristics. When applying microscopic identification methods, specific professional skills are needed and results can depend on the subjective interpretations. Small nano- and picoplanktonic cells are also difficult, if not impossible, to identify to species level (Eiler et al., 2013). These drawbacks are, for the most part, avoidable applying molecular methods for identification.

Next generation sequencing methods (NGS) enable DNA- and RNA-based analyses of uncultured species and, with exploiting the data cumulating in the data banks, biodiversity evaluation of phytoplankton can be renewed. Strong positive correlation between rRNA gene copy numbers and genome size (Prokopowich et al., 2003) or cell length in cultured algal strains (Godhe et al., 2008) gives promises for developing molecular monitoring of phytoplankton biovolumes to support and substitute microscopying. Although highly attractive, sequencing of phytoplankton samples has several challenges, which hinder the application of the tool. For phytoplankton, it is difficult to find broad-range PCR primers, and therefore primer bias can skew the actual diversity scene of microbes in community studies (Hong et al., 2009; Hadziavdic et al., 2014; Hugerth et al., 2014; Bradley et al., 2016). Another obstacle for molecular phytoplankton analysis arises from the lack of the classified sequences in the databases (Abad et al., 2016). Although several reference databases exist for rRNA genes of prokaryotes (SILVA, Greengenes, RDP) and for plastidial rRNA genes (Decelle et al., 2015) for photosynthetic eukaryotes, overall taxonomic resolution for phytoplankton is poor and scattered. As the NGS and single-cell technologies mature, we can expect expanding libraries and increasing lengths and qualities of reads, which will increase the taxonomic resolution of molecular phytoplankton analysis.

One challenge involves DNA/RNA extraction from the cells, as many comparative studies have described differences in isolation efficiencies (Stach et al., 2001; Hong et al., 2009; Simonelli et al., 2009; Rosic and Hoegh-Guldberg, 2010; Eland et al., 2012; Koid et al., 2012). Sample preservation in Lugol or by freezing, cell lysis and nucleic acid extraction without degradation are critical steps that can complicate the isolation of DNA and RNA from phytoplankton cells. Environmental samples contain cells with diverse cell properties, varying in cell size and firmness of cell walls, which may favor certain cells when using particular extraction procedures. Various physical, chemical and enzymatic cell lysis protocols are used in commercial kits, but bead-beating has become a gold standard. Yuan et al. (2015) found that bead-beating method can double the DNA yield of some phytoplankton species in comparison with the enzymatic non-bead-beating method. Eland et al. (2012) has suggested that additional freeze-thaw lysis might influence the effectiveness of beat beating. Although NGS enables molecular assessment of the diversity of microbial eukaryotic communities (Lie et al., 2014), factors like the primer bias and differences in DNA or RNA isolation efficiencies can mask the actual phytoplankton diversity and skew the results of environmental samples.

To study how sample preservation and the nucleic acid extraction methods affect NGS analysis of phytoplankton communities, we made a comprehensive experiment with a mock community comprising three algal classes (diatoms, dinoflagellates and green algae), two strains per each class. Sequencing results were compared against microscopic observations, dry masses and carbon contents of the mock cell pool. When finding that DNA-based analysis did not follow the biomass estimates we evaluated the variation in the rRNA gene copy number per DNA by using qPCR-based approaches on the separately extracted mock strains. Bioinformatics was optimized by performing the NGS sequencing for individually barcoded mock strain samples and evaluating the distribution of sequences in this model data during the steps of the trimming pipeline.

## Materials and Methods

### Study Strategy

**FIGURE 1:**
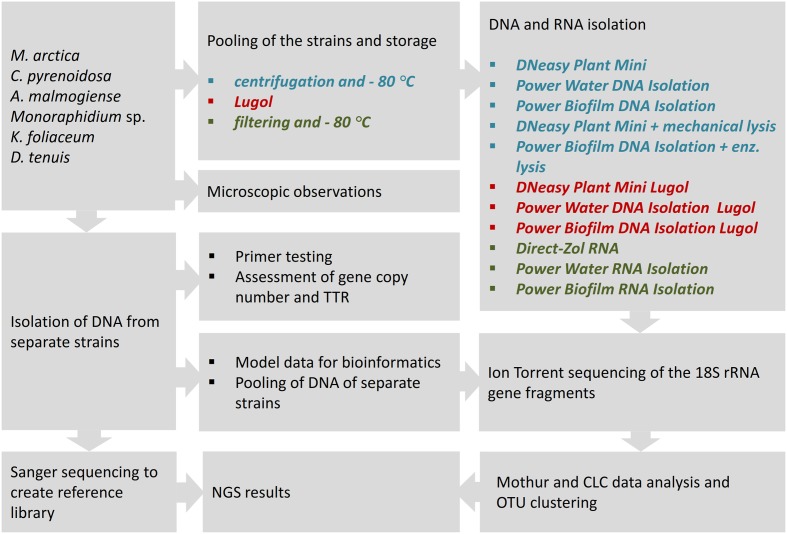
The study strategy for the NGS analysis of a mock community, comprising of six phytoplankton strains, to compare cell-preserving and nucleic acids extraction methods. For nucleic acid isolation, three DNA extraction kits with their enzymatic or mechanical cell lysing modifications, and three RNA extraction methods were applied to study cell pools, preserved at –80°C or in acidic Lugol’s solution. TTR refers to theoretical template relationship, OTU refers to operational taxonomic unit. Full strain names are presented in Section “Materials and Methods.”

In this comparative study, NGS results of the mock community pool of six phytoplankton strains were analyzed according to used sample preservation and nucleic acid extraction methods (**Figure 1**). To interpret the NGS results, DNA samples were isolated from separate strains, and reference library of the 18S rRNA gene sequences was created applying Sanger sequencing. The NGS results were compared with original cell numbers, biomass and carbon content values in the mock pool. To evaluate the reliability of the nucleic acid isolation methods, several tests were done for separate strains. The match of the selected eukaryotic primer pair was tested *in silico* against the database and *in vitro* using quantitative PCR (qPCR) with an independent primer pair. The 18S rRNA gene copy numbers per extracted DNA and per cell were determined for each strain. TTR of rRNA genes in the original cell pool was calculated using gene copy numbers from equal volumes of extracted DNA (Power Biofilm extraction) of each strain. In the other test, separately extracted DNAs (Power Biofilm extraction) were combined in equal DNA amounts, and NGS was performed as in the original protocol. This test was done to reveal the potential bias due to preferential amplification of certain ribosomal sequence types during amplification. Therefore TTR-analysis avoided competition of primers, and “pooled DNA” analysis showed theoretical results if the DNA yields (in ng) of all mock cell cultures would have been equal. For optimizing bioinformatics pipelines, the effects of trimming procedures were evaluated with separately barcoded data of mock strains.

For the nucleic acid extraction experiments, cells of the mock community were pooled and stored in Lugol or by deep-freezing, and DNA or RNA extracts were isolated using different methods. From the extracted DNA and random primed cDNA, 18S rRNA genes were amplified using eukaryotic primers. After NGS and clustering sequences into OTUs, results were aligned to the reference sequences obtained by Sanger sequencing, and strain-specific proportions of sequences, after different cell-restoring and nucleic-acid extraction methods, were compared.

### Microscopic Analysis of Phytoplankton Mock Community Strains

Non-axenic strains of 6 phytoplankton species isolated from the Baltic Sea (Hällfors and Hällfors, 1992) included *Diatoma tenuis, Melosira arctica, Apocalathium malmogiense, Kryptoperidinium foliaceum, Monoraphidium* sp., and *Chlorella pyrenoidosa*, which were obtained from the Culture Collection of the Marine Research Centre, Finnish Environment Institute (SYKE MRC)/Tvärminne Zoological Station, University of Helsinki (Supplementary Table 1).

Phytoplankton cells were stained and mounted in ProLong Diamond Antifade Mountant with DAPI (Thermo Fisher Scientific, United States). Poly-L-Lysine (Sigma–Aldrich) was used to coat the coverslips to attach the cells. Imaging of the cells was performed using Zeiss Cell Observer HS wide-field microscope, Colibri LED light source at 365 nm wavelength for DAPI, Plan-Apochromat 63x (NA = 1.4) Ph3 oil immersion and Plan-Apochromat 100x (NA = 1.46) objectives and filter set 49 (excitation 365 nm and emission 445/50 nm).

Wet volume (biomass) and cell numbers of the mock community samples were assessed using Zeiss Axio Vert.A1 epifluorescence microscope applying counting strategy described by Salmi and Salonen (2016). Dry mass and carbon content was analyzed from the deep-frozen cell pellets (next chapter), dried in tin cups for 20 h at 65°C. The dry weight was determined using Sartorius M2P and Sartorius CP2P and the carbon mass in the dry weight sample was analyzed using the Thermo Delta V stable isotope mass spectrometer.

### Preservation of the Phytoplankton Cells, Nucleic Acids Extraction and cDNA Synthesis

Before starting the mock community study, freshly grown 2 mL cell culture of each species was harvested by centrifuging at 3500 *g* for 10 min, and supernatant was removed leaving cell pellet and 100 μL of culture medium in the tubes. DNA was extracted separately from these cell pellets using Power Biofilm DNA isolation Kit according to manufacturer’s instructions (MoBio Laboratories, Inc., Carlsbad, CA, United States) to test suitability of the primers, to inspect gene copy numbers per extracted DNA and per cell, to construct reference library applying Sanger sequencing, to pool equal DNA quantities for the control DNA pool, and to produce separately barcoded model data for optimizing the trimming pipeline (details in Section “Amplification of 18S rRNA Gene Fragments and Sequencing”.)

For comparative analysis of cell preservation and DNA isolation methods, equal volumes of fresh, in the active cell growth phase growing cultures of the mock community were pooled and divided into 2 mL aliquots, which were centrifuged at 3500 *g* for 10 min to obtain 100 μL of cell-suspension, which was kept frozen at -80°C for 2 weeks. To test if storing cells in Lugol affects sequencing results, part of the pooled sample was stored in 1% acidic Lugol’s solution (final concentration) at +4°C, and 2 mL aliquots were centrifuged at 3500 *g* for 10 min to obtain 100 μL of cell-suspension before DNA extraction. Cellular DNA was extracted from frozen and Lugol preserved cells using DNeasy Plant Mini Kit (Qiagen, United States), Power Water DNA Isolation Kit (MoBio Laboratories, Inc., Carlsbad, CA, United States), and Power Biofilm DNA Isolation Kit (MoBio Laboratories, Inc., Carlsbad, CA, United States). To determine, if addition of mechanical cell destruction would improve the cell lysis and consequently DNA yield, *DNeasy Plant Mini Kit DNA + mech.* extraction was done using manufacturer’s instruction with additional mechanical treatments. Cells were exposed to extra freeze/thaw cycle by dipping them into the liquid nitrogen and disrupting cells by beat-beating at maximum vortex speed for 10 min in 0.1 mm Glass Beads Tubes (MoBio Laboratories, United States) in AP1 buffer (Qiagen). *Power Biofilm DNA Kit + enz.* DNA isolation was extended with additional enzymatic treatment, starting with inactivation of DNases by incubating cells at 75°C for 10 min (Wiame et al., 2000) and continuing incubation in Viscozyme enzyme solution (60 mg/ml) (Sigma–Aldrich) at 50°C for 1 h and after that in Proteinase K (0.5 mg/ml) (Thermo Scientific, United States) enzyme/TE-buffer (pH 8)/SDS (0.5 %) solution at 50°C for 1 h. After these additional mechanical or enzymatic treatments, isolation continued according to manufacturer’s instructions. Three replicates were performed from all isolation methods and their variations. DNA concentration was checked using Qubit 2.0 Fluorometer and dsDNA High Sensitivity Assay Kit (Life Technologies, United States).

To perform RNA based sequencing analyses, 2 mL of fresh, pooled sample (from same pool as used in DNA extractions) was filtered through 25 mm diameter and 0.22 μm pore size polyethersulfone Millipore Express PLUS Membrane Filters (GPWP02500, Millipore, United States) using 25 mm Swinnex Filter Holders (SX0002500, Millipore, United States). After filtration the membranes were directly inserted into the MoBio Glass Beads Tubes before freezing to prevent RNA degradation when starting RNA isolation, so that lysis buffer could be added to the frozen cells. Samples were frozen at -80°C without delay and kept in freezer for 3 weeks before Direct-Zol RNA Micro Prep isolation (Zymo Research, Irvine, CA, United States) and for 2 months before MoBio Power Water and Power Biofilm RNA isolations (MoBio Laboratories, Inc., Carlsbad, CA, United States). Lysis buffer was added into the bead tubes before melting the sample tubes. Procedures of Power Water RNA Isolation Kit and Power Biofilm RNA Isolation Kit followed manufacturer’s specialized instructions to co-extract small RNA fractions. Direct-Zol kit consists of spin column purification of RNA from TRIzol, which was added into bead tubes containing frozen mock sample filters. Bead tubes were vortexed at maximum speed for 1 min and centrifuged at 12000 × *g* for 1 min before supernatant collection. Because of the low RNA yield with MoBio kits, GeneJET RNA Cleanup and Concentration Micro Kit (Thermo Scientific, United States) was used to concentrate the RNA samples. RNA integrity and concentration was determined using TapeStation 2200 applying the High Sensitivity RNA ScreenTape system (Agilent Technologies, United States) and Qubit 2.0 Fluorometer applying the RNA Assay Kit (Life Technologies, United States).

The cDNA was synthesized by reverse transcription applying RevertAid First Strand cDNA Synthesis Kit’s (Thermo Scientific, United States) using random priming from 50 ng (Power Water RNA), 5 ng (Power Biofilm RNA) and 60 ng (Direct-Zol RNA) of total RNA.

### Amplification of 18S rRNA Gene Fragments and Sequencing

Two sets of 18S rRNA gene primers were tested *in silico* with the program SILVA TestPrime and *in vitro* using quantitative PCR (qPCR) to analyze whether primer pairs, Euk1A (5′-CTGGTTGATCCTGCCAG-3′) and reverse Euk516R (5′-ACCAGACTTGCCCTCC-3′) (Díez et al., 2001; Eland et al., 2012), and V8F (5′-ATAACAGGTCTGTGATGCCCT-3′) (Bradley et al., 2016) and reverse 1510R (5′-CCTTCYGCAGGTTCACCTAC-3′) (Amaral-Zettler et al., 2009) would anneal and amplify DNA sequences of mock community species. Primer pairs targeted different variable (V) regions, V1 to V3 and V8 to V9, respectively. Equal 4 ng amount of DNA extract from each strain was used as a template in separate reactions, using Bio-Rad CFX96 real time thermal cycler (Bio-Rad Laboratories) and Maxima SYBR Green/Fluorescein qPCR Master Mix (Thermo Scientific, United States) in a 25 μl reaction mixture with 0.4 μM of primers. The qPCR procedure started with an initial denaturation step at 94°C for 3 min and continued with 35 cycles of amplification (94°C for 30 s, 52°C for 1 min and 72°C for 1 min) with final extension at 72°C for 5 min. Since the M13-tail (5′-TGTAAAACGACGGCCAGT-3′) in the 5′-end of the Euk1A-forward primer was needed for sample barcoding for NGS sequencing, qPCR amplification reactions was also done with M13-Euk1A/Euk516R primer pairs to test if the tail would interfere the amplification. The Euk1A/Euk516R primers were used to prepare templates of individual strains for Sanger sequencing (Sanger et al., 1977). However, for *K. foliaceum* direct Sanger sequencing of the 18S rRNA fragment was only successful after isolating RNA, cDNA synthesis and cloning using the CloneJet PCR Cloning Kit (Thermo Scientific, United States). Sanger sequences of mock community strains were deposited in the European Nucleotide Archive (ENA) under study accession number PRJEB22147.

To study the effect of DNA extraction methods on the sequencing results, 3 ng of extracted DNA template and primer pair M13-Euk1A/Euk516R were used and the same PCR procedure was applied as above, except that PCR amplification was limited to 30 cycles. For the cDNA samples derived from the reverse transcription reaction, 2, 3, or 3 μL of cDNA of Direct-zol RNA isolation, Power Water RNA isolation, and Power Biofilm RNA isolation, respectively, was used as a template, and the amplification followed the same procedure as for DNA samples.

First PCR amplification was followed by the eight cycles of second PCR to add the barcoded sequencing adaptor IonA-M13. Barcoding of amplicons, size-trimming of the products and final Ion Torrent sequencing was done using the Ion Torrent Personal Genome Machine (PGM) as described by Mäki et al. (2016), except using the Hi-Q and Hi-Q View OT2 Kit, Hi-Q and Hi-Q View Sequencing Kit, and Ion 316v2 chip (Life Technologies).

The copy number of 18S rRNA gene was determined for each strain separately from 2 μL volume of DNA extracts (i.e., representing equal volumes of original cultures when pooled) to predict the theoretical template relationships in the mock pool (TTR). DNA extracts were used as a template and Euk1A/Euk516R as primers in the qPCR reaction, and copy numbers were determined with duplicate 5-point standard series of mock community member PCR products ranging from 1.5 × 10^4^ to 1.5 × 10^8^ (amplification efficiency 85%, y-intercept 41 cycles). For creating model data for optimizing the NGS data trimming pipeline, each strain was amplified separately with unique barcodes, applying the same procedures as above. When an equal number (pM) of the barcoded amplicons from each strain was used in subsequent sequencing, any observed biases in abundances can be assumed to have resulted from post-PCR steps: sequencing and/or sequence analysis. To check the effect of primer bias, variation in the gene copy numbers and theoretical results, if the DNA yields of all mock cell cultures would have been equal, 4 ng of isolated DNA from each strain was pooled, amplified, barcoded, and sequenced with the same reagents and procedures as above. Amplification products were analyzed using Agilent 2200 TapeStation system with the High Sensitivity D1000 ScreenTape (Agilent, United States) and in the agarose gel electrophoresis prior to sequencing. All control analyses were done in triplicate.

### Data Analysis

The model data was utilized for evaluating and optimizing the trimming procedure. PGM sequencing data was initially trimmed with Torrent Suite 5.0.4 software including default adapter removal and adjusted polyclonality filtering (command: “--mixed-first-flow = 120 --mixed-last-flow = 160”) because of the long internal adaptors. Default 3′ end quality trimming of Torrent Suite can be turned off (command: “--trim-qual-cutoff 100”), so both 3′ end trimmed reads and reads without 3′ end trimming were imported into Mothur v.1.36.1 (Schloss et al., 2009) and CLC Genomics Workbench 9.5.1 software^1^. The trimming workflows of Mothur and CLC software were evaluated using the separately barcoded 18S rRNA gene data of mock strains, pooled in equimolar concentrations (see the trimming parameter in results, Optimizing the bioinformatics pipeline). The de novo OTU clustering in Mothur v.1.36.1 was performed using average neighbor algorithm and in CLC using distance-based greedy algorithm UCLUST (Supplementary Tables 4, 5).

The trimming pipeline that best preserved the original relationships among barcode bins was chosen for further analyses. In this protocol PGM reads were first processed using Torrent Suite 5.0.4 software without 3′ end quality trimming. After initial processing of the reads, fastq files were imported into CLC software where the quality trimming was performed according the parameters gained from the model data analysis at OTU 97% identity clustering level (Supplementary Table 4).

Relative abundances of strains were square-root transformed before calculation of Bray-Curtis similarity matrix, based on which non-metric multidimensional scaling (NMDS) was calculated with 1000 repeats in PRIMER v. 6.1.12 and PERMANOVA+ v.1.0.2 (PRIMER-E/Quest Research Limited, Albany, New Zealand).

## Results

### Phytoplankton Cells

**FIGURE 2:**
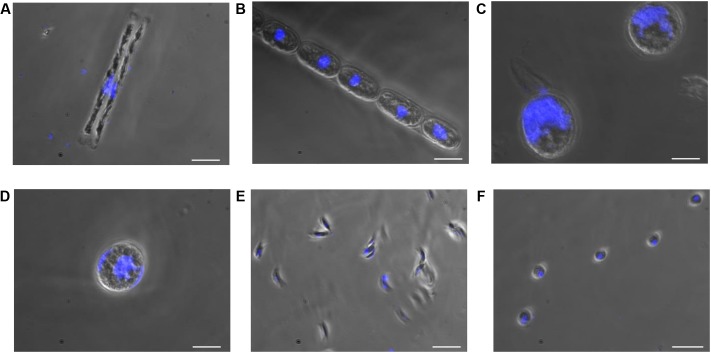
Nucleus and cell sizes of phytoplankton cells **(A)** *D. tenuis*, **(B)** *M. arctica*, **(C)** *A. malmogiense*, **(D)** *K. foliaceum*, **(E)** *Monoraphidium* sp., and **(F)** *C. pyrenoidosa* were compared using microscope for visualization. DNA of the cells was stained with DAPI. Scale bars, 20 μM.

In this study, cultures of *D. tenuis* and *M. arctica* (Diatomophyceae), *A. malmogiense* and *K. foliaceum* (Dinophyceae), *Monoraphidium* sp. and *C. pyrenoidosa* (Chlorophyceae) phytoplankton cells (Supplementary Table 1) were observed using a light microscope to determine the biomass, cell number (Supplementary Table 2), and the location and size of nucleus. Nuclei sizes varied between 2 and 28 μm among the species, being largest in *A. malmogiense* and *K. foliaceum* cells (**Figure 2**). A second nucleus of a diatom endosymbiont was visible in the dinoflagellate *K. foliaceum* (**Figure 2D**). NGS results confirmed the purity of the cultures and specificity of the primers used in the study, as 98% (variation 93–100%) of the NGS sequences could be classified to the six target strains when strains were sequenced separately or in the mock community pool (Supplementary Figure 1A). Only the data of dinoflagellate *K. foliaceum* contained 14% non-target sequences, which were derived from the known endosymbiont nucleus of diatom origin (Figueroa et al., 2009).

### Amplification and Sequencing of the Partial 18S rRNA Gene

**FIGURE 3:**
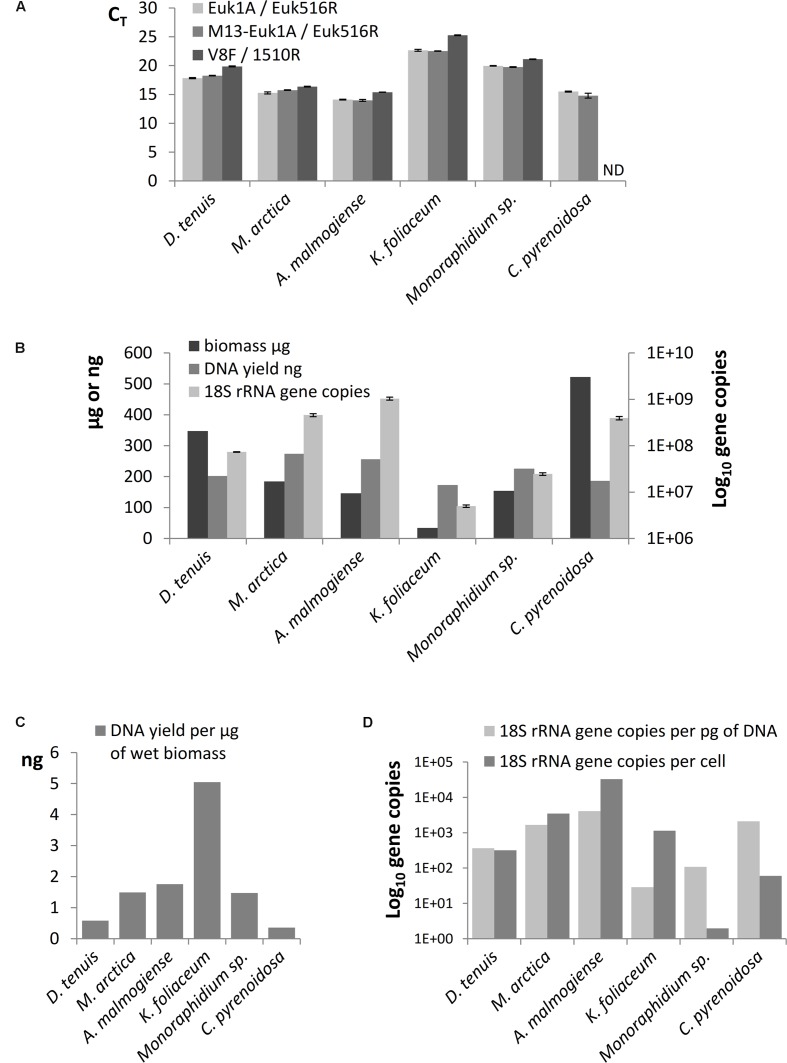
**(A)** Comparison of threshold cycles (C_T_) of qPCR when amplification of 18S rRNA genes was performed using Euk1A/Euk516R, M13-Euk1A / Euk516R and V8F / 1510r primer pairs from equal quantity of genomic DNA (4 ng). **(B)** Total wet biomasses, DNA yields, and 18S rRNA gene copy numbers per Power Biofilm extraction. qPCR results are presented as mean values of triplicates with standard errors. ND indicates the sample that was not possible to amplify with the V8F/1510R primer pair. **(C)** DNA yields per ng of wet biomass. **(D)** 18S rRNA gene copy numbers per cell and per pg of extracted DNA.

The results of SILVA TestPrime test (Supplementary Table 3) indicated that the Euk1A F / Euk516R primer pair was appropriate for amplification of fragments of the 18S rRNA gene of all six phytoplankton species. The threshold cycles (C_T_) of qPCR of separately extracted mock strain DNAs confirmed that Euk1A/Euk516R primer pair amplified 18S rRNA gene of all species and M13-adapter part in forward primer did not affect the amplification efficiency (**Figure 3A**). Although the qPCR results with V8F/1510r primer pair mostly corresponded to C_T_ values of the other primer pair, this pair only amplified 5 of the 6 study species (not *C. pyrenoidosa*). 18S rRNA gene copy numbers in the extracted DNA were determined from the qPCR performed with the Euk1A / Euk516R primer pair (**Figure 3B**). The results showed 100-fold differences in the rRNA gene numbers in the mock strain DNAs, without correlation to original biomasses or DNA yields. *K. foliaceum* had the highest DNA yield per biomass (**Figure 3C**). Calculated ribosomal copy numbers per cell varied between 2 in *Monoraphidium* sp. to 33 000 in *A. malmogiense* (**Figure 3D**), which means over 10^4^ variation in the rRNA operons per cell among the study strains.

### Optimizing the Bioinformatics Pipeline

**FIGURE 4:**
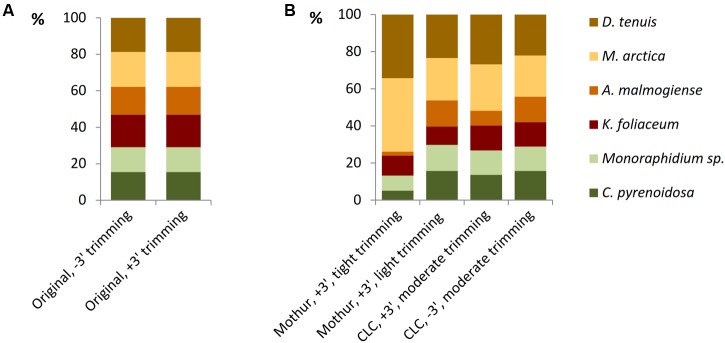
The effect of trimming criteria on the NGS results of the model data of separately barcoded sequences of mock strains. **(A)** Torrent Suite 3′ end trimming of reads (+3′) retained the original relationship of barcoded reads as well as without trimming (–3′) imported reads. **(B)** When 3′ end trimmed (+3′, in Torrent Suite) reads were imported into the Mothur software and tight trimming procedures were applied, proportions of the strains became distorted. Resolving trimming lighter, proportions appeared more equal. The pipeline without 3′ end trimming in Torrent Suite and moderate trimming in CLC was selected for further analyses.

The model data of separately barcoded mock strain sequences was collected to optimize the quality trimming pipeline. 3′ end trimmed reads and reads without 3′ end trimming were imported from Torrent Suite 5.0.4 software to Mothur or CLC software and, before further trimming, strain-specific proportions of reads were equal in both the data sets (**Figure 4A**). When using the Mothur software for 3′ end trimmed reads and imposing tight quality requirements, such as minimum length of 180 bases and minimum quality average of 20 over a sliding window of 10 nucleotides (Supplementary Table 5), considerable number of *A. malmogiense* sequences were trimmed off and excessive increase of *M. arctica* sequences was observed (**Figure 4B**). When trimming requirements in Mothur were relaxed, with minimum length of 150 bases and no sliding-window quality check, proportions of sequences were less biased. The other trimming processes in both cases were kept similar, including in maximum two allowed mismatches in the primer region, one mismatch in the barcode region, and the maximum homopolymer length of eight. In the CLC pipeline, when reads without 3′ end trimming were imported from Torrent Suite and minimum length of read was imposed to 150, proportions of sequences followed the original distribution better than when starting with 3′ end trimmed reads. More careful examination of trimming revealed that the site that induced temporary decrease in the quality values was a loop in the rRNA gene structure. In the CLC program the default modified-Mott quality algorithm was used for end-trimming with error probability limit of 0.05. In both software programs, OTU_0.97_ clustering was applied to identify similar sequences and the OTUs were then classified to species level against the reference library (Supplementary Tables 4, 5). When all OTU sequences, gained from nucleic acids extraction methods and from studies of separately sequenced strains, were aligned against the reference sequences of the mock community, target sequences were the most prevalent of all OTUs (Supplementary Figure 1 and Table 6). Although 98% of the OTU_0.97_ sequences could be classified to right phytoplankton strains, comparison of rarefaction curves of the whole data at OTU_0.97_ and OTU_0.99_ levels showed that RNA extractions yielded less small sequencing errors than DNA extractions, which may be attributed to less PCR cycles needed for amplification of rRNA genes (Supplementary Figures 1B,C). CLC trimming settings retained the original distribution of sequences and therefore that pipeline was used for further comparison of data from the nucleic acid extraction methods.

### Nucleic Acid Yield and NGS Results of the Mock Pool

**FIGURE 5:**
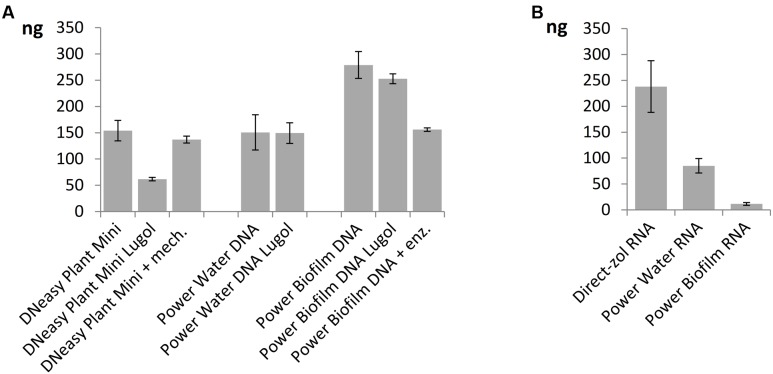
Yields of nucleic acids from the mock phytoplankton community using **(A)** three DNA extraction kits with or without Lugol preservation or extra enzymatic or mechanical cell lysis steps and **(B)** three RNA extraction kits. All but Lugol stored samples were preserved at -80°C. Total extracted DNA or RNA yields are presented as mean values of three isolations with standard errors.

When comparing nucleic acid extraction methods, highest DNA yields of the mock community was gained with Power Biofilm DNA isolation kit (**Figure 5A**). Additional mechanical lysis steps, here freeze/thaw cycle and beat-beating, or additional enzymatic lysis method, here incubation in Viscozyme/Proteinase K, did not increase the DNA yield when DNeasy Plant Mini Kit or Power Biofilm DNA isolation kit, respectively, were used and when compared to standard protocols recommended by the manufacturer. Lugol preservation decreased the DNA yield of the DNeasy extraction, but did not significantly affect the yield of the other kits. When comparing RNA extraction procedures the highest overall yield and preservation of small RNAs was gained using TRIzol-based Direct-zol RNA isolation method (**Figure 5B**). Size distribution histograms of final RNA extracts illustrate the integrity of RNA after extraction methods, showing best performance by the Direct-zol kit (Supplementary Figure 2).

**FIGURE 6:**
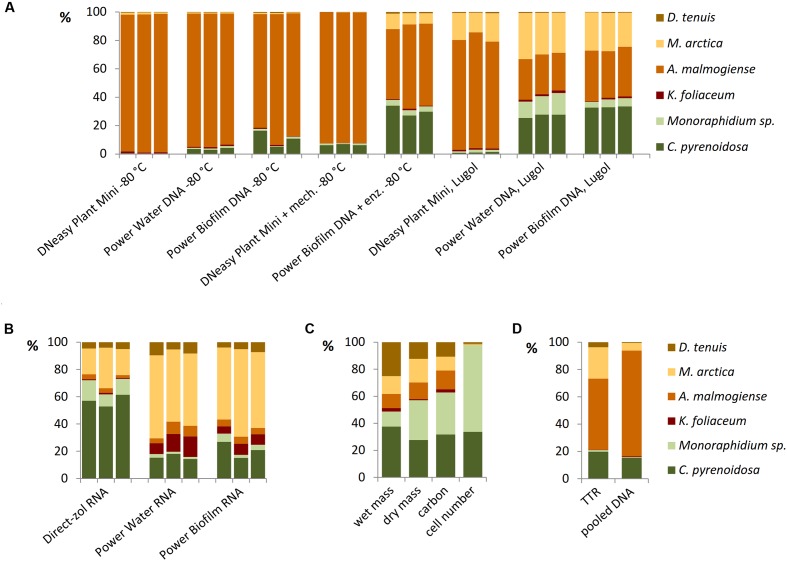
Relationships of mock community strains based on **(A)** 18S rRNA gene analyses of DNA extractions and **(B)** 18S rRNA analyses of RNA extractions, **(C)** microscopically determined wet biomasses and cell numbers and dry mass and carbon content determinations, and **(D)** theoretical results based on separately extracted DNA’s of mock strains using Power Biofilm kit. TTR refers to theoretical template relationship, when DNA extracts of strains were isolated and amplified separately counting the results based on individual 18S qPCR results, and pooled DNA refers to a sample containing equal amounts (weight) of DNA of mock strains.

Different DNA extraction and RNA extraction methods strongly affected the NGS results of the mock pool (**Figures 6A,B**). When Power Water and Biofilm kits, based on mechanical cell lysis (bead-beating), were used, Lugol preservation brought out green algae species better than when cells were preserved at -80°C (**Figure 6A**). Results of microscopic biomass counting (**Figure 6C**) showed that although small green algae species were numerically dominating in the mock pool, biomass values appeared quite evenly distributed, except *K. foliaceum*. (**Figure 6C** and Supplementary Table 2). Wet and dry biomass, cell carbon content, TTR values and NGS results of separately extracted and equally pooled DNA (**Figures 6C,D**) were used as indicators to evaluate different nucleic acid extraction methods. Strain specific copy numbers of 18S rRNA gene, determined using the qPCR, were used to calculate the theoretical template relationships in the original pool (TTR).

**FIGURE 7:**
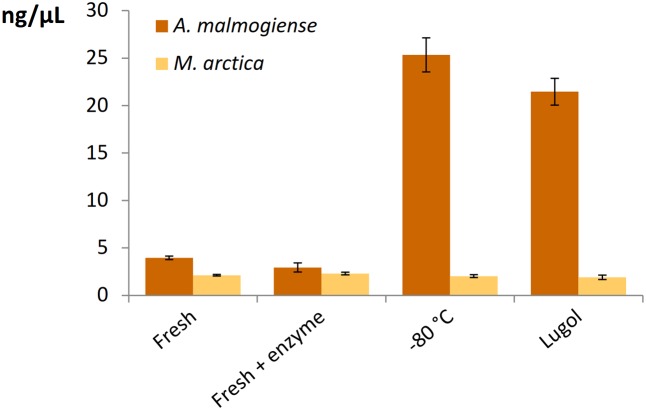
The effect of storage and additional enzymatic lysis steps on the DNA yields of *A. malmogiense* and *M. arctica*. DNA was extracted using Power Biofilm kit from fresh cells with or without additional enzymatic step or from cells stored at –80°C or in Lugol.

All the DNA isolation methods, except samples with additional enzymatic cell lysis, demonstrated that the *A. malmogiense* sequences strongly dominated the data if DNA extraction was done from frozen cells (**Figure 6A**). Preserving cells in Lugol, and usage of additional enzymatic lysis step favored green algae and *M. arctica* species decreasing *A. malmogiense* sequences, when Power kits were in use. Sequences of *K. foliaceum* were very weakly amplified from DNA isolations compared to RNA based sequencing (**Figures 6A,B**). Since *A. malmogiense* was overrepresented in all at -80°C stored DNA samples, and proportion of *M. arctica* increased in the Lugol preserved samples, we did additional DNA extractions using Power Biofilm isolation kit. We wanted to see if storing conditions and additional enzymatic lysis would affect the DNA yield of these two species. In this test additional enzymatic lysis steps did not affect the yield of *M. arctica* or *A. malmogiense* DNA extractions from fresh cells, but promoted a tenfold increase in DNA yields of *A. malmogiense* when the samples were stored at -80°C or in Lugol (**Figure 7**) before DNA extraction.

**FIGURE 8:**
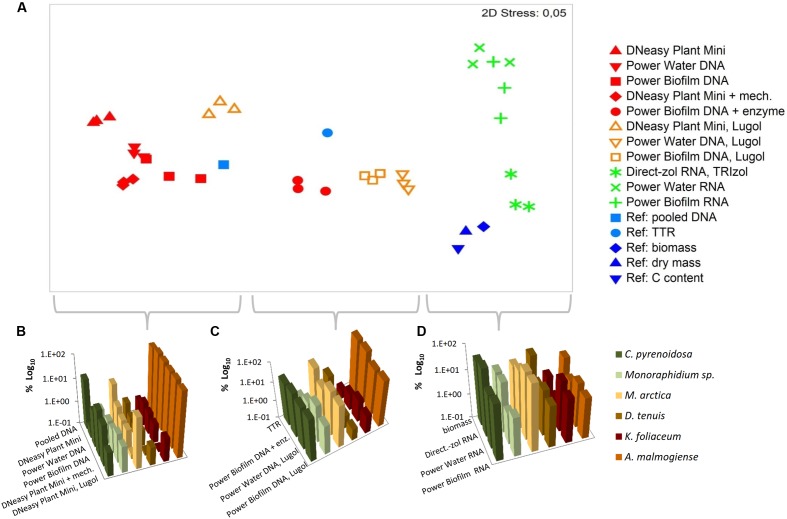
Comparison of numerical relationships of mock species based on the NGS results and reference parameters. **(A)** Non-metric multidimensional scaling (NMDS) ordination of the data. **(B–D)** Illustration of the data in subsets separated by the NMDS ordination. TTR refers to theoretical template relationship. Note that the percentages in the y-axis are shown in logarithmic scale.

Based on the NMDS ordination, DNA-based analyses (samples stored at -80°C), Lugol-preserved DNA-based analyses and RNA-based analyses were separated on the primary (horizontal) axis (**Figure 8A**). In this set, RNA-based analyses (especially Direct-zol extraction) most closely resembled the biomass (**Figures 8A,D**), dry mass and carbon content proportions of the mock cell pool. DNA-based NGS results were mostly affected by the high gene copy numbers of *A. malmogiense*, which overpowered the abundance of other species (**Figures 8A,B**). When the DNA samples were preserved with Lugol, this effect was not as massive, and the data resembled more RNA results, as well as biomass, dry mass and carbon content results (**Figures 8A,C**). The difference between deep-frozen and Lugol-preserved samples was large, even if the concentrations of the DNA extractions were in similar level. Sequence abundances of RNA extraction samples was the best indicator of biomass and, using Direct-Zol RNA isolation method, the presence of both green algae species was remarkable, as also in the biomass calculation (**Figure 8D**).

## Discussion

Molecular methods, especially high-throughput sequencing, have shown their effectiveness in the study of diversity and ecology of phytoplankton, potentially replacing traditional microscopic identification and quantification methods. Recently, the massive Tara Oceans voyage surveyed 210 ecosystems at global scale applying NGS methods and collecting environmental data (de Vargas et al., 2015; Pesant et al., 2015). The data of the expedition provided profound knowledge on eukaryotic plankton species revealing that their diversity was wider than earlier expected. Using NGS methods, indeed, it is possible to detect rare taxa when other identification techniques might miss these (Yu et al., 2015). Wang et al. (2014) have defined two critical genetic factors, which affect the results of molecular- and OTU-based characterization studies. At first, genetic polymorphisms of eukaryotic microscopic organisms are still unknown, which makes a point of defining OTUs at an optimal dissimilarity level. Another factor is that while bacterial genomes have only from one to several 16S rRNA gene copies, eukaryotic genomes may have thousands of 18S rRNA genes. Proper interpreting of rRNA gene-based abundances has crucial role in molecular characterization of protists, whose rRNA gene copy numbers can vary from a few in small species to 100s of 1000s in large species like dinoflagellates and ciliates (Fu and Gong, 2017), and actually in this study the variation spanned from 2 to 33 000 rRNA operons per cell. When interpreting NGS results of environmental samples, species with small nucleus and low gene copy numbers may be hidden in cases when a sample is rich with high gene copy number species, as here by *A. malmogiense*. Our study showed that small nucleated diatoms, even if having a high total biomass when compared to other species, displayed only minor occurrence in the final NGS results. This was due to lower rRNA gene copy numbers per DNA, as differences within the primer match was excluded by determining the gene copy numbers using two qPCR primer pairs targeting independent conservative areas of the ribosomal RNA gene.

The DNA yields from the mock community strains were on average 0.2% of the wet biomass when isolated using the Power Biofilm DNA isolation kit. When testing the other DNA extraction kits and their modifications on the mock pool, highest overall DNA yield was gained with Power Biofilm DNA isolation kit, and the yield was not improved by additional enzymatic lysis steps. Highest RNA yields (and also best small RNA yields) were obtained by the Direct-zol extraction system.

The overall yield of nucleic acids does not necessary indicate the best extraction method, if the quantitative diversity of species is subject of the study. Here, for example, some methods gave similar yields, but NGS results appeared different. This was especially clear between samples preserved at -80°C and in Lugol. Lugol preservation tended to decrease the dominance of the dinoflagellate *A. malmogiense* that was most efficiently extracted from deep-frozen samples, actually much better than from fresh samples even with the Power Biofilm kit. When adding an additional enzymatic lysis step for the Power Biofilm kit, DNA yields did not change, but the proportion of *A. malmogiense* decreased, giving way to diatom and green algae sequences.

To find out how should the most realistic mock community NGS data look out and evaluate studied nucleic acid extraction methods the results were portrayed against biomass and dry mass values, as well as qPCR-based analyses of the TTR and pooled DNA sample. Our results showed some correlation between theoretical prediction (TTR) and with Lugol preserved Power Water and Biofilm DNA isolation values or with Power Biofilm DNA isolation with additional enzymatic cell lysis method values. Wet biomass was considered to be the most natural indicator to which sequencing results could be compared, since it is the method that has been in use in traditional limnology and oceanology. Our results demonstrate that while DNA-based methods were mostly affected by the rRNA copy number variation, results based on random primed cDNA as a starting material yielded the most realistic measures of the biomass values.

As described in reviews published by Robasky et al. (2014) and van Dijk et al. (2014), NGS data can be easily biased in many phases over the procedures during the library preparation. Also our results demonstrated that many different factors influence the NGS results, but furthermore, data trimming can cause additional bias when certain sequences are discriminated. NGS data quality trimming must be customized to suit the study and sequencing platform. For example, gentle trimming of NGS data of low PHRED scores have been suggested (MacManes, 2014) and for RNA-seq trimming, justification of caution exist (Williams et al., 2016). Evaluation of bioinformatics steps can also be done using *in silico* sequence libraries, although they do not replace the real sequencing data (Hardwick et al., 2017). We suggest that a control sample of few known species, relevant to the study in question, should be included into the NGS, and the effects of the data trimming should be followed through the pipeline. In this study it was convenient to evaluate trimming effects using separately barcoded sequences. In the Ion Torrent sequencing, it is possible that secondary structures (loops) of the certain rRNA genes structures may have delayed the sequencing signal, thus temporarily decreasing the quality value, which later increased to the normal level. Whether this can be possible when sequencing with other platforms is not known by us.

Although many challenges still exist in molecular level identification of phytoplankton species like sequence data analyzing issues, primer biases and imperfection of DNA and RNA extraction methods, the advantages of molecular methods go beneath the surface. One remarkable benefit is that the data obtained from studies can be utilized in the long term when tools and capacity for bioinformatics data continue to develop. Considering phytoplankton molecular identification tools, one obstacle is the lack, limitedness or inaccuracy of reference libraries. The data collected beforehand can be reanalyzed and completed when libraries have been extended.

Deeper characterization of community structure of phytoplankton has advanced through new NGS techniques and tools for data interpretation are continuously improving (Johnson and Martiny, 2015) but evaluation of methods is still needed. Even though rare species may be revealed from the data, quantitative assessment of data may turn out excessively demanding. Microscopic observation, flow cytometry studies and other tools of identification and quantification of phytoplankton cells have proved their utility values in the past and are important tools to validate NGS results. This study showed that RNA-based data better correlated with biomass parameters and, as it indicates active protein synthesizing capacity of the community, avoids the problems of possible relic DNA (Carini et al., 2016).

## Conclusion

We present thus far one of the most complete comparison of microscopic and molecular analysis of phytoplankton communities with real biomass and carbon values, especially focusing on the effects on the selection of nucleic acid extraction methods. This study demonstrated that DNA-based phytoplankton analysis was principally affected by the huge rRNA gene copy number variation among phytoplankton species, which makes quantitative NGS studies of phytoplankton very challenging to interpret. In the light of this study, it is possible and even favorable to preserve phytoplankton samples deep-frozen before extraction procedures. Preserving the samples in acidic Lugol’s solution resulted in equal DNA yields and PCR performance, but affected community profiles. When comparing traditional biomass values and sequencing results, none of the DNA-based extraction methods resulted in coherent data, but RNA-based methods yielded more realistic relationship of organisms. Finally, the study demonstrated that bioinformatics can form a post-laboratory bias, if sequences are cut with narrow sliding-window algorithms, since the data quality has sequence-specific variation in the sites of secondary structures.

## Author Contributions

Study was designed by AM, PS, and MT. PS counted phytoplankton samples and carried out dry mass and carbon content analysis. AM performed microscope imaging, molecular biology experiments, NGS and data analysis. AMi assisted with bioinformatics and performed statistical analysis. Manuscript was prepared by AM. All authors contributed to the discussion of the results, reviewed and edited the manuscript. AK offered important intellectual knowledge about phytoplankton cells.

## Conflict of Interest Statement

The authors declare that the research was conducted in the absence of any commercial or financial relationships that could be construed as a potential conflict of interest.

## Footnotes

1 www.qiagenbioinformatics.com

## Abbreviations

TTR: theoretical template relationship
