# Supplementary material for: Sample Preservation, DNA or RNA Extraction and Data Analysis for High-Throughput Phytoplankton Community Sequencing
Source: Front Microbiol. 2017 Sep 26;8:1848. doi: 10.3389/fmicb.2017.01848 (PMC5622927; doi:10.3389/fmicb.2017.01848)
Supplement: Supplementary file 1 [file Data_Sheet_1.pdf]

*Supplementary Material*

# Sample preservation, DNA or RNA extraction and data analysis for high-throughput phytoplankton community sequencing

**Anita Mäki[1*,] Pauliina Salmi[1], Anu Mikkonen[1], Anke Kremp[2] and Marja Tiirola[1]**

[1]Department of Biological and Environmental Science, University of Jyväskylä, Jyväskylä, [2]Marine Research Centre, Finnish Environment Institute, Helsinki, Finland

*** Correspondence:**
Anita Mäki
anita.maki@jyu.fi

## Supplementary Figures

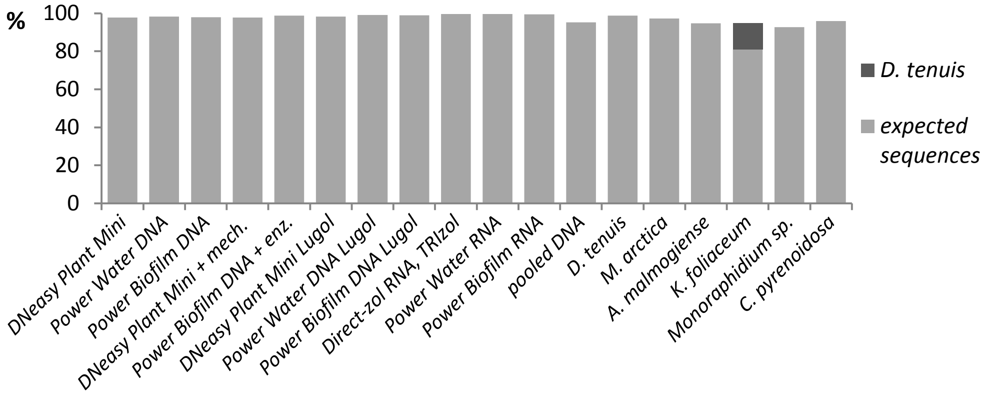


A

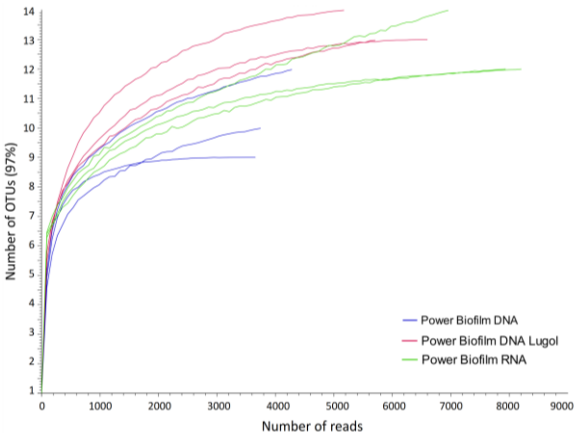


B

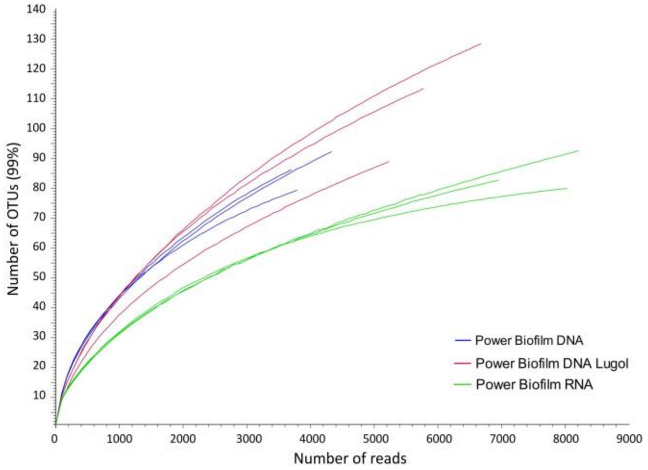


C

**Supplementary Figure 1.** Percentage of the sequences in the six main OTUs (similarity 0.97), that represent the six target phytoplankton strains and examples of rarefaction curves. (A) The average covery of these six main OTUs was 98 % (93–100 %) of sequences. Among the separately tested species was an exception, *K. foliaceum*, which is a binucleate cell having additional nucleus and 18S rRNA gene of diatom origin. (B) Example rarefaction curves of NGS results using $OTU_{0.97}$ clustering and (C) using $OTU_{0.99}$ clustering.

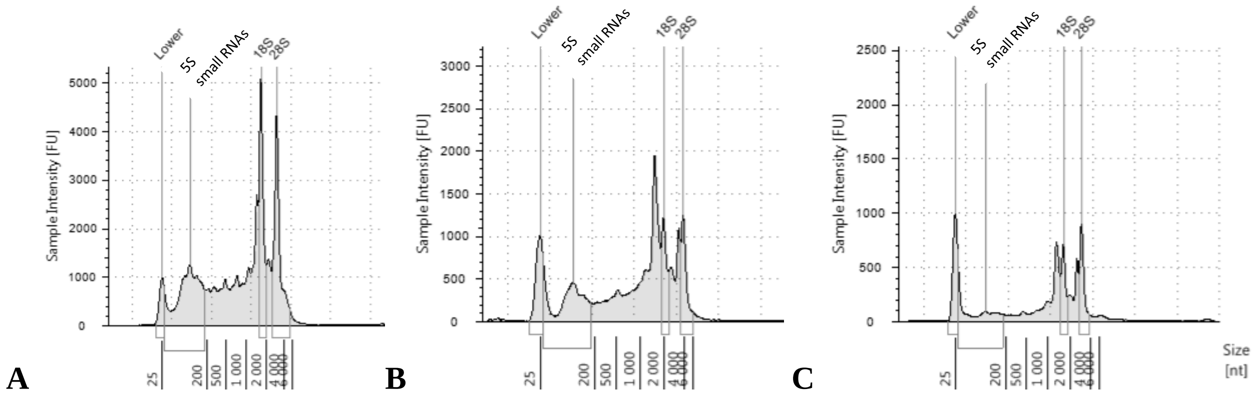


**Supplementary Figure 2.** Size distribution histograms of RNA extractions using (A) Direct-zol RNA extraction, (B) Power Water RNA isolation, and (C) Power Biofilm RNA isolation. The extracts were analyzed using TapeStation 2200 and the High Sensitivity RNA ScreenTape. Lower marker designates the 25 nt peak size.

## Supplementary Tables

**Supplementary Table 1.** Algal strains of which the mock community pool was comprised for DNA and RNA isolation.

| Strain ID | Taxon | Location of isolation | Time of isolation | Isolated by |
|---|---|---|---|---|
| SHTV-1 | *Apocalathium malmogiense* | Tvärminne/Storfjärden | 2002 | Anke Kremp |
| KFF-1001 | *Kryptoperidinium foliaceum* | Åland/Föglö | 2010 | Päivi Hakanen |
| DTTV-1401 | *Diatoma tenuis* | Tvärminne/Storfjärden | 2014 | Päivi Hakanen |
| MATV-1402 | *Melosira arctica* | Tvärminne/Längden | 2014 | Johanna Oja |
| TV70 *) | *Monoraphidium* sp. | *) | *) | *) |
| TV216 *) | *Chlorella pyrenoidosa* | *) | *) | *) |

*) (Hällfors G, and S Hällfors, 1992)

**Supplementary Table 2.** Light microscopy data of the phytoplankton cell cultures before pooling the cells for nucleic acids isolation.

| | *Diatoma tenuis* DTTV-1401 | *Melosira arctica* MATV-1402 | *Apocalathium malmogiense* SHTV-1 | *Kryptoperidinium foliaceum* KFF-1001 | *Monoraphidium* sp. TV 70 | *Chlorella pyrenoidosa* TV216 |
|---|---|---|---|---|---|---|
| medium | 6 psu f/2 +Si | 6 psu f/2 +Si | 6 psu f/2 +Si | 6 psu f/2 +Si | 6 psu f/2 +Si | 6 psu f/2 +Si |
| growth temperature (°C) | 4 | 4 | 4 | 16 | 16 | 16 |
| inoculation date | 3.11.2015 | 3.11.2015 | 3.11.2015 | 3.11.2015 | 3.11.2015 | 3.11.2015 |
| Preparation of samples for microscopy | 1 mL culture + 2.5 mL PBS + 7.5 µL Lugol | 1 mL culture + 2.5 mL PBS + 7.5 µL Lugol | 1 mL culture + 2.5 mL PBS + 7.5 µL Lugol | 1 mL culture + 2.5 mL PBS + 7.5 µL Lugol | 250 µL culture + 3.25 mL PBS + 7.5 µL Lugol | 250 µL culture + 3.25 mL PBS + 7.5 µL Lugol |
| Abundance in culture (cells $L^{-1}$) | 115197805 | 65894078 | 15821197 | 2185918 | 6264498945 | 3278874126 |
| Abundance in culture (cells $m^{-3}$) | 1.15198E+11 | 65894078000 | 15821197000 | 2185918000 | 6.2645E+12 | 3.27887E+12 |
| Abundance in culture (cells $mL^{-1}$) | 1.15E+05 | 6.59E+04 | 1.58E+04 | 2.19E+03 | 6.26E+06 | 3.28E+06 |
| Biomass (mg $m^{-3}$) | 173805.4575 | 92110.6282 | 72934.4289 | 17145.8941 | 76877.9311 | 261099.6395 |
| Biomass (µg $mL^{-1}$) | 173.8054575 | 92.1106282 | 72.9344289 | 17.1458941 | 76.8779311 | 261.0996395 |
| CFL95% for biomass | 29 | 29 | 29 | 34 | 4 | 18 |
| Parallel fields counted | 16 | 20 | 12 | 50 | 10 | 10 |
| Average cell mass (mg) | 1.51E-06 | 1.40E-06 | 4.61E-06 | 7.84E-06 | 1.23E-08 | 7.96E-08 |
| Average cell volume (µL) | 1.51E-06 | 1.40E-06 | 4.61E-06 | 7.84E-06 | 1.23E-08 | 7.96E-08 |
| Average cell volume (1 fL = 1 1 $µm^3$) | 1509 | 1398 | 4610 | 7844 | 12 | 80 |
| Cell shape | cylinder | circular cylinder | flattened ellipsoid | flattened ellipsoid | double cone | sphere |
| Average cell lenght (µm) | 60.5 | 16.3 | 25.3675 | 29.87 | 7.5 | - |
| Lenght range (µm) | 57.5-75 | 12.5-37.5 | 20.0-30.0 | 15.0-37.5 | - | - |
| Average cell width (µm) | 5.0 | 10.3 | 19.9225 | 23.93 | 2.5 | 5.23125 |
| Width range (µm) | - | 10.0-15.0 | 15.0-25.0 | 12.5-30.0 | - | 5.0-7.5 |
| Cell depth/height (µm) | 5.0 | - | 17.1375 | 18.4938 | - | - |
| depth range (µm) | - | - | 12.5-20.0 | 10.0-25.0 | - | - |

**Supplementary Table 3.** The match of the Euk1A F / Euk516 R primer pair for mock community taxons tested using the TestPrime tool against the non-redundant version of the SILVA SSU Ref database allowing one mismatch occurrence.

| taxonomy | coverage | specificity | accessions | eligible | match | mis-match | no-data |
|---|---|---|---|---|---|---|---|
| Eukaryota;Archaeplastida;Chloroplastida;Chlorophyta;Chlorophyceae;Sphaeropleales;Monoraphidium; | 100 | 92.5 | 15 | 5 | 5 | 0 | 10 |
| Eukaryota;Archaeplastida;Chloroplastida;Chlorophyta;Trebouxiophyceae;Chlorellales;Chlorella; | 88.9 | 92.5 | 16 | 9 | 8 | 1 | 7 |
| Eukaryota;SAR;Alveolata;Dinoflagellata;Dinophyceae;Peridiniphycidae;Peridiniales;Kryptoperidinium; | 100 | 92.5 | 2 | 2 | 2 | 0 | 0 |
| Eukaryota;SAR;Alveolata;Dinoflagellata;Dinophyceae;Peridiniphycidae;Thoracosphaeraceae;*)Scrippsiella; | 88.9 | 92.5 | 90 | 9 | 8 | 1 | 81 |
| Eukaryota;SAR;Stramenopiles;Ochrophyta;Diatomea;Bacillariophytina;Bacillariophyceae;Diatoma; | 100 | 92.5 | 10 | 6 | 6 | 0 | 4 |
| Eukaryota;SAR;Stramenopiles;Ochrophyta;Diatomea;Coscinodiscophytina;Melosirids;Melosira; | 66.7 | 92.5 | 6 | 3 | 2 | 1 | 3 |

*) *Scrippsiella hangoei* is an earlier synonym of *A. malmogiense*, see Craveiro, S. C., Daugbjerg, N., Moestrup, Ø., & Calado, A. J. (2017). Studies on *Peridinium aciculiferum* and *Peridinium malmogiense* (=*Scrippsiella hangoei*): comparison with *Chimonodinium lomnickii* and description of *Apocalathium gen. nov*. (Dinophyceae). *Phycologia*, *56*(1), 21-35. DOI: 10.2216/16-20.1.

**Supplementary Table 4.** Final trimming and OTU picking parameters for the NGS data using CLC Genomics Workbench 9.5.1 software. For the comparative testing of nucleic acid extraction methods a total of 362,728 sequences were processed, of which 136,778 sequences were removed during the trimming.

| Trimming and OTU picking parameters | |
|---|---|
| Trim adapter list | M13_Euk1A |
| Quality trim | Yes |
| Quality limit | 0.05 |
| Minimum number of nucleotides in reads | 150 |
| OTU picking | De novo OTU clustering |
| Similarity percentage | 97 % |
| Minimum occurrences | 10 (2 in “posit. control”) |
| Fuzzy match duplicates | No |
| Find best match | Yes |
| Chimera crossover cost | 3 |
| Kmer size | 6 |

**Supplementary Table 5.** Tested pipeline for the model data analysis using Mothur v.1.36.1 bioinformatics platform. Applying quality criteria for trimming, such as minimum length of 180 bases and minimum quality average over a window of 20, resulted in biased proportional sequence abundances of model data sample and was not used for final data analysis. The commands used and the number of usage of CPUs are presented according the order they were assigned. The dataset was named as "phyto" and "summary.seqs" command was given frequently to follow the processing.

| A brief comment of the function of trimming | Command in Mothur software |
|---|---|
| Extract sequences reads from a .sff file | sffinfo(sff=phyto.sff) |
| Preprocess features needed to screen and sort sequences | trim.seqs(fasta=phyto.fasta, oligos=phyto3.oligos, qfile=phyto.qual, pdiffs=2, bdiffs=1, maxambig=0, maxhomop=8, qwindowaverage=20, qwindowsize=10, minlength=180, processors=16) |
| Unique (re-replicate) identical sequences to save time in processing | unique.seqs(fasta=phyto.trim.fasta) |
| Align a fasta-formatted sequences against Silva database | align.seqs(fasta=phyto.trim.unique.fasta, reference=silva.nr_v123.align, flip=T, processors=8 |
| Summarize the quality of sequences (e.g. check the start and end points for the next command) | summary.seqs(fasta=phyto.trim.unique.align, name=phyto.trim.names) |
| Fulfill or cull defined criteria | screen.seqs(fasta=phyto.trim.unique.align, name=phyto.trim.names, group=phyto.groups, start=1046, optimize=end, criteria=95, processors=8) |
| Remove columns from alignments based on a defined criteria | filter.seqs(fasta=phyto.trim.unique.good.align, vertical=T, trump=., processors=8) |
| Unique identical sequences | unique.seqs(fasta=phyto.trim.unique.good.filter.fasta, name=phyto.trim.good.names) |
| Remove sequences for sequencing error mitigation | pre.cluster(fasta=phyto.trim.unique.good.filter.unique.fasta, name=phyto.trim.unique.good.filter.names, group=phyto.good.groups, diffs=2) |
| Search for chimeric sequences | chimera.uchime(fasta=phyto.trim.unique.good.filter.unique.precluster.fasta, name=phyto.trim.unique.good.filter.unique.precluster.names, group=phyto.good.groups, processors=16) |
| Remove chimeric sequences | remove.seqs(accnos=phyto.trim.unique.good.filter.unique.precluster.denovo.uchime.accnos, fasta=phyto.trim.unique.good.filter.unique.precluster.fasta, name=phyto.trim.unique.good.filter.unique.precluster.names, group=phyto.good.groups, dups=T) |
| Classify sequences taxonomically against database using defined criteria | classify.seqs(fasta=phyto.trim.unique.good.filter.unique.precluster.pick.fasta, name=phyto.trim.unique.good.filter.unique.precluster.pick.names, group=phyto.good.pick.groups, template=silva.nr_v123.align, taxonomy=silva.nr_v123.tax, cutoff=80, iters=1000, processors=16) |
| Generate a new file that contains sequences of defined taxon (excluding removed) | remove.lineage(fasta=phyto.trim.unique.good.filter.unique.precluster.pick.fasta, name=phyto.trim.unique.good.filter.unique.precluster.pick.names, group=phyto.good.pick.groups, taxonomy=phyto.trim.unique.good.filter.unique.precluster.pick.nr_v123.wang.taxonomy, taxon=unknown) |
| Rename all the filenames into a simplified format (example: only fasta-file) | system(cp phyto.trim.unique.good.filter.unique.precluster.pick.pick.fasta phyto.final.fasta) |
| Calculate pairwise distances between | dist.seqs(fasta=phyto.final.fasta, cutoff=0.15, processors=16) |

| | |
|---|---|
| aligned DNA sequences so OTU clustering can be done accordingly | |
| Assign sequences to OTUs (default clustering algorithm: average neighbor) | cluster(column=phyto.final.dist, name=phyto.final.names) |
| Create a OTU-file, OTUs occurrence per barcode | make.shared(list=phyto.final.an.list, group=phyto.final.groups, label=0.03) |
| Classification of OTUs | classify.otu(list=phyto.final.an.list, name=phyto.final.names, taxonomy=phyto.final.taxonomy, label=0.03) |
| Generates a fasta-file containing only a representative sequence for each OTU | get.oturep(column=phyto.final.dist, list=phyto.final.an.list, name=phyto.final.names, fasta=phyto.final.fasta, method=abundance, weighted=true) |

**Supplementary Table 6.** Clustering of the NGS reads to target phytoplankton strains ($OTU_{0.97}$ level) of the mock community pool and separate cell cultures. *K. foliaceum*, which is known to be a binucleate containing nucleus of diatom origin and accordingly had two dominant sequences of 18S rRNA gene.

| Extraction method or strains | All reads | Target sequences | Target sequences % of all reads |
|---|---|---|---|
| DNeasy Plant Mini | 10655 | 10421 | 97.8 |
| Power Water DNA | 10112 | 9939 | 98.3 |
| Power Biofilm DNA | 11891 | 11654 | 98.0 |
| DNeasy Plant Mini + mech. | 11344 | 11090 | 97.8 |
| Power Biofilm DNA + enz. | 14764 | 14589 | 98.8 |
| DNeasy Plant Mini Lugol | 11676 | 11479 | 98.3 |
| Power Water DNA Lugol | 16527 | 16395 | 99.2 |
| Power Biofilm DNA Lugol | 17652 | 17468 | 99.0 |
| Direct-zol RNA, TRIzol | 23112 | 23016 | 99.6 |
| Power Water RNA | 20921 | 20828 | 99.6 |
| Power Biofilm RNA | 23192 | 23074 | 99.5 |
| pooled DNA | 21657 | 20627 | 95.2 |
| *D. tenuis* | 26595 | 26250 | 98.7 |
| *M. arctica* | 27189 | 26455 | 97.3 |
| *A. malmogiense* | 17321 | 16403 | 94.7 |
| *K. foliaceum*<br>*(D. tenuis)* | 19345 | 15652<br>(2683) | 80.9<br>(13.9) |
| *Monoraphidium* sp. | 16834 | 15604 | 92.7 |
| *C. pyrenoidosa* | 19609 | 18800 | 95.9 |
